# The sterilizing effect of pyriproxyfen on the malaria vector *Anopheles gambiae*: physiological impact on ovaries development

**DOI:** 10.1186/s12936-015-0609-3

**Published:** 2015-03-04

**Authors:** Bayili Koama, Moussa Namountougou, Roger Sanou, Sévérin Ndo, Abdoulaye Ouattara, Roch K Dabiré, David Malone, Abdoulaye Diabaté

**Affiliations:** Institut de Recherche en Sciences de la Santé/Centre Muraz, Bobo-Dioulasso, Ouagadougou, Burkina Faso; Universite Polytechnique de Bobo, Bobo-Dioulasso, Ouagadougou, Burkina Faso; Innovative Vector Control Consortium, Liverpool, UK

**Keywords:** Malaria, Anopheles gambiae, Pyriproxyfen, Insecticide, Bed net, Resistance, Ovaries, Fecundity, Fertility, Inhibition

## Abstract

Adult females *An. gambiae* were exposed in 3 min cone test to treated nets with PPF before or after they were blood fed. The effects of PPF on ovaries development, females oviposition and eggs hatching were assessed. Both unfed and fed mosquitoes exposed to PPF exhibited nearly complete inhibition of fecundity (70-100%) and fertility (90-100%). After females have been exposed once to PPF, the sterilizing effect on their fecundity was observed over 3 consecutive blood meals suggesting that PPF might have an irreversible sterilizing effect. Observation of the ovaries of exposed females to PPF under microscope revealed that the ovaries failed to develop even after several blood meals. The combination of PPF to pyrethroids on bednets could provide better malaria control tool and prevent the further development and spread of pyrethroid resistance in malaria vectors.

## Background

Pyrethroid insecticides are the most widely used compounds in public health because of their high effectiveness and strong excito-repellent effect on insects, as well as low mammalian toxicity [1-3]. Unfortunately resistance to this insecticide class has become widespread in malaria vectors in several malaria endemic countries and that may challenge in the future the success of malaria control programmes [4-7]. Clearly alternative compounds that can complement pyrethroids on long-lasting insecticidal nets (LLINs) are urgently needed. This approach can provide an opportunity to preserve the protectiveness of LLINs through the excito-repellent properties of the pyrethroids while enhancing toxicity through a non-pyrethroid alternative [8,9]. Pyriproxyfen (PPF), an insect juvenile hormone mimic, is recommended by the World Health Organization (WHO) [10,11] for larval control but may also have an impact on life expectancy, fecundity and fertility of adult mosquitoes through tarsal contact. PPF has an extremely low toxicity to humans and shows no cross resistance to other classes of insecticides used in public health [11]. PPF is an insect growth regulator used primarily to inhibit metamorphosis of mosquito larvae and prevent emergence of adults from pupae [12,13], hence its use for mosquito control has been limited to larval stages [14-16]. However, studies have shown that PPF can affect the development and production of eggs (fecundity) and reduces their hatching (fertility) [17,18]. Earlier studies on *Aedes* mosquitoes have shown that exposure to PPF can reduce the reproductive capacity of adults [19-21] depending on dosage and time of exposure in relation to the blood meal [19]. A complete sterilization of females was observed with *Anopheles gambiae* exposed to PPF-treated netting [22] and *Anopheles arabiensis* exposed to PPF in bottle bioassays [23]. More recently, a mixture of PPF and pyrethroids on bed nets achieved a strong sterilizing effect on pyrethroid-resistant *An. gambiae s.s.* in experimental hut trials [9].

Whilst most studies have focused on evaluating the raw outcomes of PPF on female mosquito fecundity and fertility, none has ever looked at the specific changes operated on the ovaries. Notably, no study has investigated to the best of current knowledge the ability of PPF to interfere with the development of ovaries in mosquitoes (but see Brabant and Dobson [24]). The current study explores this gap and assesses for the first time the physiological impact of PPF on *An. gambiae* Kisumu female ovaries when exposed to PPF treated nets at different times after blood meals in the laboratory.

The following hypotheses were tested:

1. PPF has an irreversible structural damage on ovaries and females can no longer lay eggs (over multiple gonotrophic cycles) after being in contact with the chemical once;
2. When the ovaries are fully developed and females have laid eggs (parous) before any contact with PPF, subsequent contact with the chemical has only minor effect on successive ovipositions.

## Methods

### Mosquitoes

The study was carried out in the laboratory of IRSS/Centre Muraz in Burkina Faso. *Anopheles gambiae* Kisumu strain was reared to adult level under standard controlled conditions (26 ± 2°C, 80 ± 10% RH and 12:12 l-D) in the insectary where larvae were fed with Tetramin™ baby fish food every day. Pupae were collected from tubs and transferred into holding cages measuring 30×30×30 cm covered with mosquito netting. Upon emergence, males and females (100:100) were maintained in cages on 5% sugar solution for five days to allow insemination. On day 5, about ten females were extracted from each holding cage and their spermathecae were dissected under a microscope to look at the insemination rate. Insemination rate exceeded 90% in most situations and the females could proceed for the cone tests. Mosquitoes were fed on a rabbit for oviposition.

### Cone test

**Figure 1:**
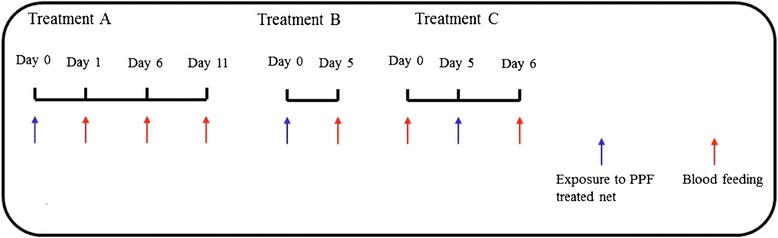
**Description of treatments to test the following hypotheses: 1) PPF has an irreversible structural damage on ovaries and females can no longer lay eggs (over multiple gonotrophic cycles) after being in contact once with the chemical (Treatments A and B); 2) When the ovaries are fully developed and females have laid eggs (parous) before any contact with PPF, subsequent contact with the chemical has only minor effect on successive ovipositions (Treatment C).**

A bioassay test was carried out with a PPF (1%)-treated net using WHO cone bioassay. The nets used in this study were made of polyethylene filaments, and proved by Sumitomo Chemical, Japan. Bioassays were conducted with non blood-fed adult female mosquitoes as recommended by the WHO [14]. Batches of ten to twelve mosquitoes were exposed for three minutes to PPF-treated nets. Overall 6–7 replicates were done per test and 4–5 tests performed. Control batches of mosquitoes were exposed for the same time to untreated nets. After exposure, mosquitoes were transferred into insecticide-free observation cages (15cm×15cm×15cm) and maintained on 5% sucrose solution at a temperature ranging from 25 to 28°C. The following three treatments were tested (see Figure 1, inspired by Harris *et al.* [23]):

### Treatment A: females exposed to PPF-treated nets and sterilization effect assessed on three subsequent blood meals

Batches of five mosquitoes (previously non blood-fed, five days old) were transferred into the cones and were exposed for three minutes to PPF-treated nets. After exposure, mosquitoes were transferred into insecticide-free observation cages containing an oviposition cup and were provided with the opportunity to blood-feed on rabbit. After the subsequent five days following the blood meal, the number of eggs laid was counted daily. Thereafter, females that survived were transferred into clean cages containing an oviposition cup and were fed again on rabbit for another oviposition cycle and this cycle was done three times. The eggs were transferred on filter papers and counted under a binocular microscope then retransferred into their original oviposition cup containing 125 mL of water for eggs hatching. The number of larvae that hatched from the eggs laid was also counted manually with a pipette and recorded on a daily basis up to five days after the first larva was seen. These were compared to a control group of Kisumu females that were exposed to untreated nets. In each cycle of oviposition, ten females were extracted at twenty four and thirty six hours after blood meal from both the exposed and non-exposed groups to PPF-treated nets and dissected to look at the ovary development; a photographic record was made of the dissected ovaries.

### Treatment B: females exposed to PPF-treated nets four days before blood meal

Unfed batches of five mosquitoes (five days old) were transferred into the cones and were exposed for three minutes to PPF-treated nets. After exposure, mosquitoes were transferred into insecticide-free observation cage for four days without any blood meal, but maintained on 5% sucrose solution. On day 5, after continuous sugar meal feeding, mosquitoes were provided with the opportunity to blood-feed on rabbit and oviposition cups were provided. A control group exposed to untreated nets had the same treatment. The number of eggs and larvae were recorded on subsequent days as described above and compared to the control group.

### Treatment C: females exposed to PPF-treated nets after a first blood meal and an oviposition cycle completed

Five days old females fed on rabbit were transferred per ten in cages (15cm×15cm×15cm) and provided with oviposition cups. The number of eggs and larvae was subsequently recorded. After oviposition, batches of five mosquitoes were transferred into the cones and were exposed for three minutes to PPF treated nets and another batch to untreated net. Twenty-four hours after exposure, mosquitoes were transferred per ten into insecticide-free observation cage (containing an oviposition cup) and were given the opportunity to blood-feed on rabbit. The number of eggs laid by females as well as the number of larvae that hatched were counted as described above and compared to that of a control group.

### Ovary dissection

In an attempt to look at the impact of PPF on ovary development batches of ten specimens from both the control and PPF-exposed groups in treatment A were extracted twelve hours and thirty six hours after the blood meal and dissected. Females were anesthetized in the freezer (−20°C) for five to ten minutes. Then they were individually dissected under a binocular in a drop of distilled water by pulling out the last segment of the abdomen. The extracted ovaries were mounted under a coverslip and observed at 200× magnification under a microscope. A fine scale observation was done at 400× magnification for a focus view on egg development. The pictures of the ovaries were taken using a digital camera looking through the eyepiece lens.

## Data analysis

In all cases fecundity was measured as the total number of eggs/total number of females that contributed to the oviposition. Similarly fertility was calculated by dividing the total number of larvae/total number of females that contributed to oviposition. The reduction of fecundity and fertility was compared between the group exposed to PPF-treated net and the control group using non-parametric Mann–Whitney tests.

## Results

### Females exposed to PPF-treated nets and sterilization effect assessed on three subsequent blood meals

**Figure 2:**
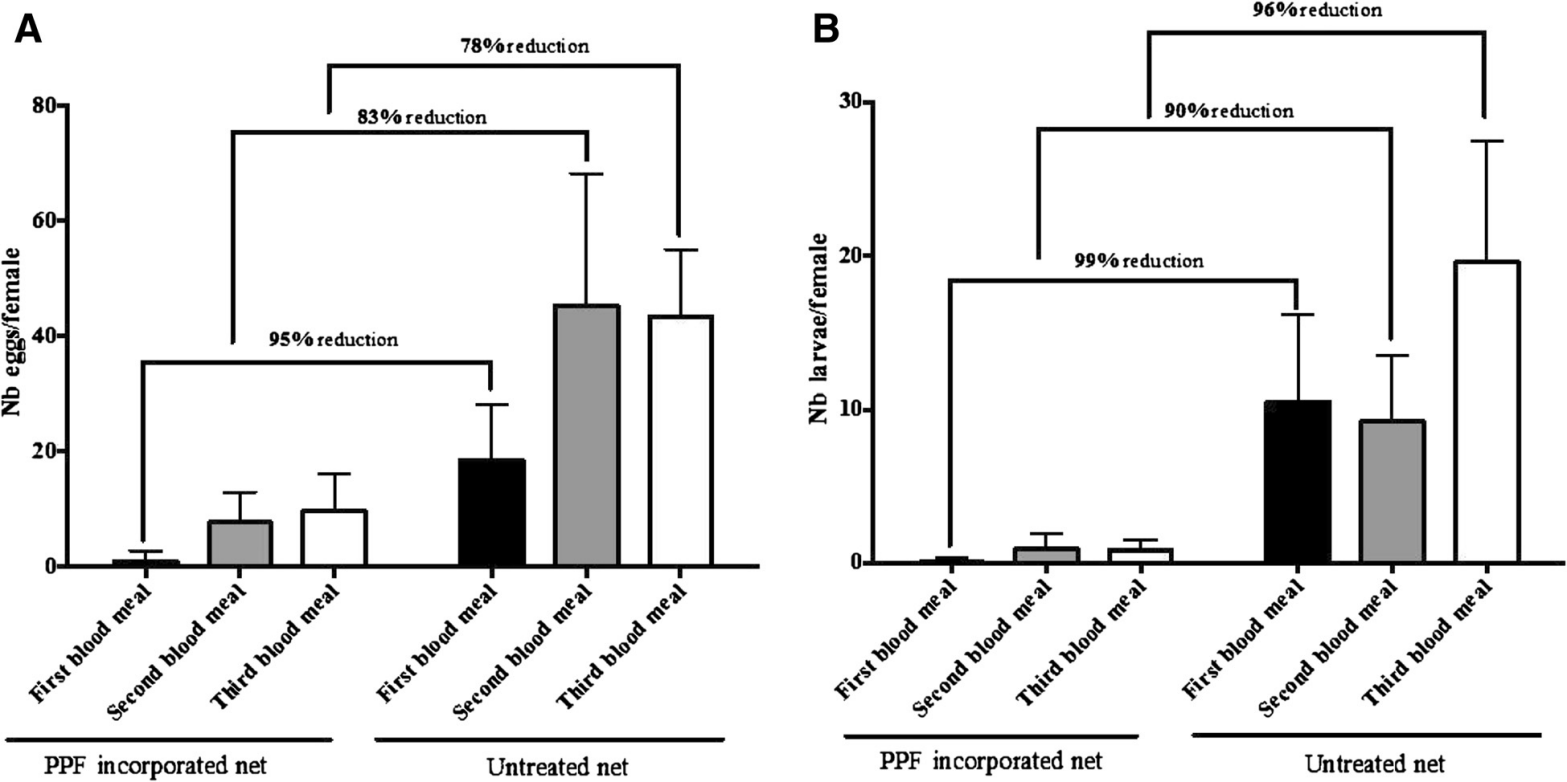
**Reduction of fecundity and fertility of female A** ***nopheles gambiae*** **sequentially exposed to 3 blood meals.** Mean number of eggs laid **(A)** and larvae **(B)** per female in control vs PPF-exposed groups. Females were exposed to treated net at day 0 and the sterilization effect of the PPF was assessed on 3 subsequent oviposition cycles.

Overall 5,046 eggs from 160 females and 1233 eggs from 338 females were recorded respectively in the control and the exposed groups to PPF-treated nets over the three cycles of oviposition. When reported as the number of females that contributed to the egg laying in each group, a significant reduction in female fecundity was observed in the PPF group (Figure 2A, Mann Whitney test P < 0.0001). Although the exposed group after the first contact had no longer been in contact with the treated nets, a significant reduction in fecundity was still observed after the second and third blood meals (Figures 2A, Mann Whitney test P < 0.0001). Reduction in fecundity was very strong in the first and subsequent cycles of oviposition and overall ranged from 78 to 95%. Similarly a very strong impact on fertility was observed from the first blood meal to the third one and reduction ranged from 90 to 99% (Figure 2B, Mann Whitney test P < 0.0001). Overall egg hatching in the control group ranged from 25 to 50% and was low in the treated group ~15%.

### Females exposed to PPF-treated nets four days before blood meal

**Figure 3:**
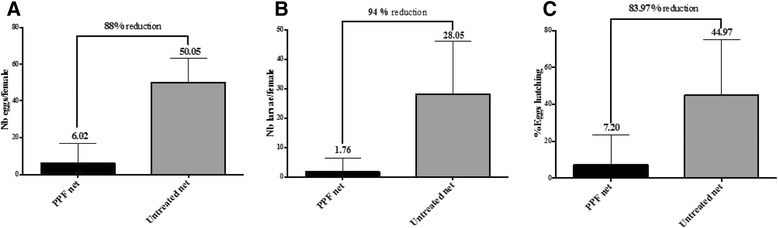
**Residual activity of PPF on** ***Anopheles gambiae*** **female fecundity and fertility exposed to PPF 4 days before blood meal.** Mean number of eggs laid **(A)** and larvae **(B)** per female in control vs PPF exposed groups. Females were exposed to PPF at day 0 and continuously fed on sugar meal until day 4. At day 5 they were blood fed and the sterilizing effect of PPF assessed. Proportion of eggs hatched in the PPF group compared to the control group **(C)**.

Figure 3A shows the mean number of eggs produced by females exposed to PPF-treated nets four days before any blood meal. Overall 578 eggs were produced by 96 females in the group exposed to PPF while 2653 eggs were produced by the control group (53 females). A significant decrease (88%) in the mean number of eggs laid per female was observed between the PPF group and the control (Mann Whitney test P < 0.0001, Figure 3A). Similarly the mean number of larvae produced per female decreased from 28 larvae/female to 1.8 larvae/female, a reduction of 94% (Mann Whitney test P < 0.0001, Figure 3B). A substantial reduction (94%) in the proportion of eggs that hatched was observed as well between the two groups (Figure 3C).

### Females exposed to PPF-treated nets after a first blood meal and an oviposition cycle completed

**Figure 4:**
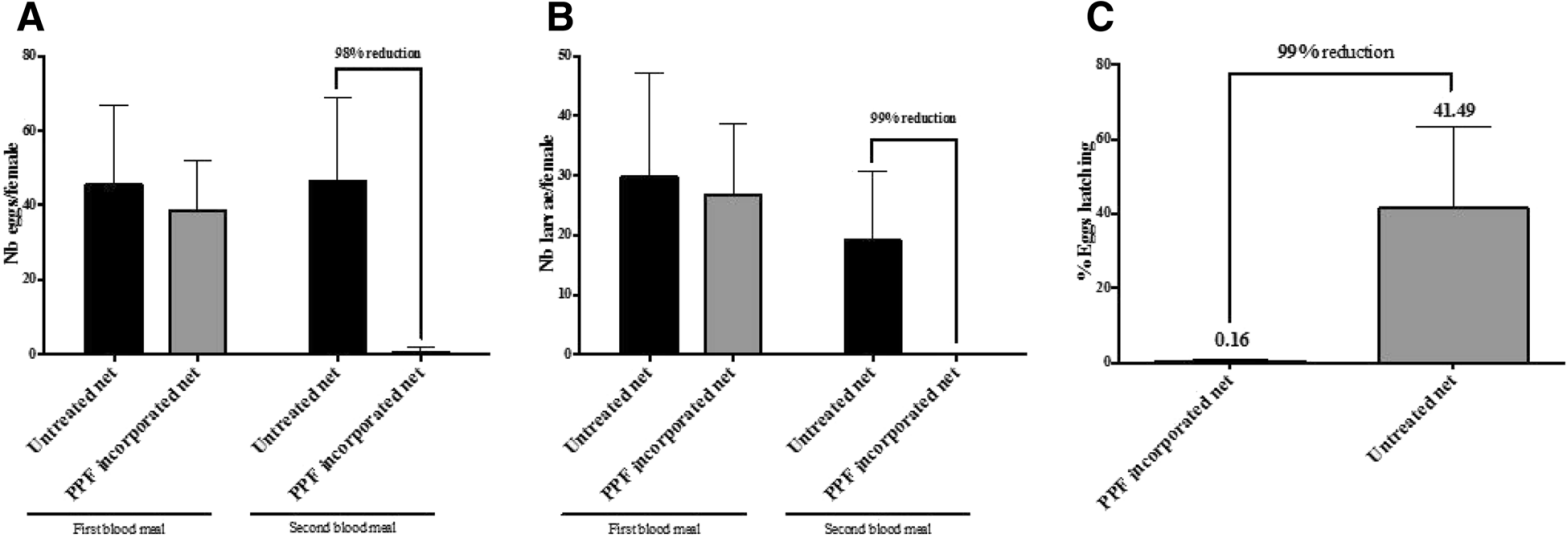
**Impact of PPF on** ***Anopheles gambiae*** **female fecundity and fertility after a first full gonotrophic cycle has been completed.** Mean number of eggs laid **(A)** and larvae **(B)** per female in the control vs PPF-exposed groups. The so called PPF group was first exposed to untreated net like the control group, then blood fed and underwent a first normal cycle of oviposition. Females of this PPF group were subsequently exposed to treated nets and blood fed a second time to assess whether PPF could still impact egg laying and hatching when a female has gone through at least one normal gonotrophic cycle. Proportion of eggs hatched in the PPF group compared to the control group **(C)**.

Figure 4A showed the mean number of eggs produced per female before and after exposure to PPF treated nets. Overall 4653 eggs were produced by 110 females in the control group, while 103 eggs were produced by 210 females in the group exposed to PPF. Before exposure to PPF, both groups after the first blood meal produced approximately an equal number of eggs (Mann Whitney test P = 0.55). However females exposed to PPF treated nets produced significantly fewer eggs (98% reduction) after the second blood meal (Mann Whitney test P < 0.0001, Figure 4A). A significant decrease in the number of larvae/female was observed as well (Mann Whitney test P < 0.0001, Figure 4B) and the proportion of eggs hatched decreased from 26.73% in the control group to 0.004% in the treated group, a reduction of 99% (Figure 4C).

### Physiological impact on ovary development

**Figure 5:**
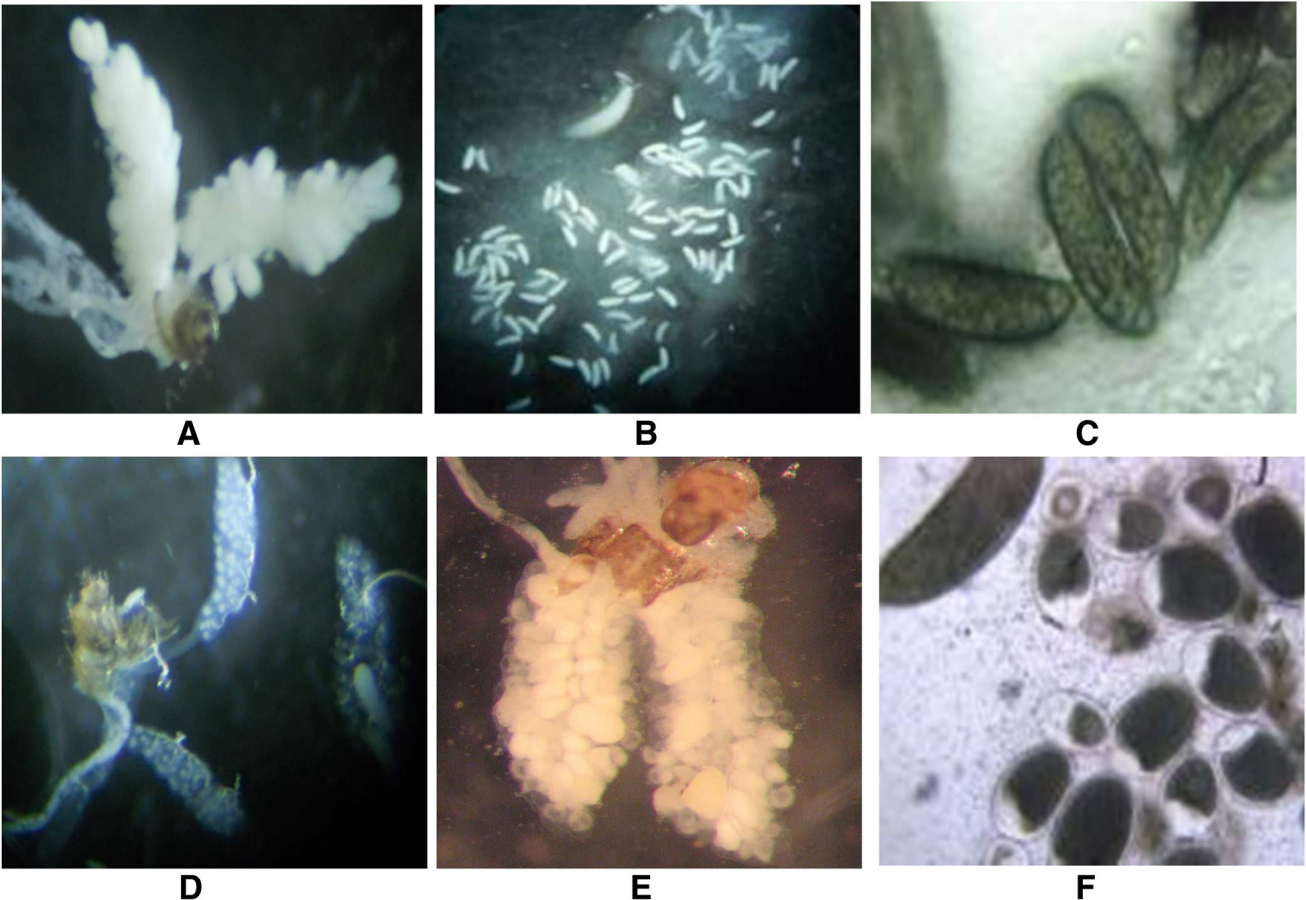
**Effect of PPF on the ovaries development.** Picture of ovaries of *An. gambiae* 12 hours after blood meal **(A)** and of egg fully formed showing the sausage shape thirty six hours after blood meal **(B and C)** in the control group. **D**, **E** and **F** are the exact replicates of **A**, **B** and **C** but in the PPF-exposed group. Note that **A**, **B**, **D** and **E** were shot at 200× magnification, while **C** and **F** were done at 400× magnification.

Overall 120 females were dissected for the ovary observation, of which 60 females belonged to the control group (thirty females at twelve hours after blood meal and 30 females at thirty six hours after blood meal) and the remaining sixty belonged to the PPF-exposed group. As eggs at a late stage (thirty six hours after blood meal) were already shaped and fully developed, hence extending the ovaries, all attempts to get the ovaries intact in the control group were vain. Though the observation of the ovaries in both control and PPF-exposed group was done at the same magnification (200× for Figure 5A, B and D, E), the ovaries in the control group were broken and the eggs released. Clear differences in term of the developmental stage of the ovaries could be seen between the control and PPF-exposed groups at both the early and late stages after blood feeding (twelve and thirty six hours after blood meal), but morphological differences of the eggs could be noticed only at the later stage of the egg development (i.e., thirty six hours after blood meal). While all the 60 females of the control group had fully developed eggs at thirty six hours after blood meal, only 12 females in the PPF-exposed group had completed the level of development as seen in the control one, however with very few eggs, some of which were aborted (Figure 5F).

Figures 5 showed the normal development of eggs in ovaries in an early developmental phase (twelve hours after the blood meal 5A) and at a later stage where eggs were fully developed showing the sausage shape (thirty six hours after the blood meal, 5B) in females that were not exposed to PPF treated nets. Figure 5C and D are the exact replicates of 5A and B however in females that were exposed to PPF-treated nets, showing a complete failure of eggs at the later stage to develop into mature eggs. Normal egg at thirty six hours after blood meal was defined as the one fully developed, elongated and showing the sausage shape (5B). Abnormal eggs were less elongated and rather exhibited a spherical shape (Figure 5D).

## Discussion

The effect of PPF on fecundity and fertility has been investigated in several insects but the mechanisms by which PPF interferes with female reproductive outcomes has been overlooked specifically in mosquitoes. The main goals in the present study were to describe the physiological impact of PPF on ovarian development and explore the sterilizing effects of PPF at various times before blood meals.

The results of the present study provided further evidence that tarsal contact with PPF-treated nets sterilized adult females of *An. gambiae* under laboratory conditions. Importantly, bioassays analysis showed that PPF impaired the development of eggs in the ovaries of *An. gambiae*, one of the most important malaria vectors in many part of sub-Saharan Africa. Exposure to PPF affected the rate at which ovarian follicles develop from the previtellogenic resting stage to maturity. Females of *Anopheles* require blood meals to mature their eggs. Females emerge from the larval stage with their primary follicles at stage I and need a blood meal to complete follicle development to stage V. In the present study, three minutes exposure of female *An. gambiae* to PPF-treated nets halted follicle maturation as they never reached stage V. Christophers [25] divided the course of ovarian development of *Anopheles* into five stages (I to V), based on the appearance of follicles under the microscope; it is now possible to relate Christophers stages to some of the physiological events of oogenesis. Several external factors influence development toward these stages in mosquitoes including nutrition [26]. Large well nourished females are able to develop their primary follicles to the previtellogenic resting stage without blood-feeding, therefore at the expense of reserves accumulated during larval life. Most wild *An. gambiae* need two blood meals to develop their first batch of eggs [26]. In females of *Aedes aegypti* that take a small blood meal, the development of follicles arrests in stage III but resumes development after a further blood meal [27,28]. In the present study, female *An. gambiae* were given full blood meals but still could not lay eggs after being in contact with PPF-treated nets. Juvenile hormone has important functions in the previtellogenic phase, stimulating development of stage I follicles to the previtellogenic stage and inducing the fat body to become competent to synthetize vitellogenin. PPF, a juvenile hormone analogue, may not initiate these functions and that might disrupt these hormonal routes leading to a failure of egg development. Further juvenile hormone level in the mosquito blood stream consistently drops after blood meal and that initiates the secretion of ecdysone by the ovary. Once released, the ecdysone directs the development of eggs ([24] and references therein). It is suspected that in the present case, PPF, the analogue juvenile hormone, was still highly present in PPF exposed females blood stream, inhibiting the release of ecdysone. Methoprene, another mimic juvenile hormone, was shown in a recent study to significantly reduce the length of ovary and the size of oocyte in *Ae. aegypti* females [24]. In the current study, a fine scale observation of the egg development showed that after each cycle of oviposition, follicles in the ovarioles of females not exposed to PPF underwent successive developmental stages known as Christopher developmental stages. Follicles were all in the same developmental stage (Christopher stage II) and deposit of yolk could be seen in the ovum at the base of the follicles. In contrary in females exposed to PPF-treated nets, the follicles were very slow to develop and never reached the complete maturation stage and showed different developmental stages. Some were still in the early developmental stage (Christopher stage II) with a round structure, while some were elongated and dark, a sign of egg abortion.

That females exposed to PPF-treated nets could not lay eggs after the first blood meal but also on subsequent blood meals, indicates the long, residual activity of this chemical on *An. gambiae* reproduction. In addition, mosquitoes exposed to PPF and blood-fed five days later could not lay eggs. Mosquitoes that had undergone a first oviposition cycle and were then subsequently exposed to PPF could no longer lay eggs. Most of the few eggs laid in certain cases did not hatch suggesting that either the larvae failed to develop or they died after development has been completed. These results are in apparent contradiction to those of Harris *et al.* [23] who did not show a long residual activity of PPF. It should be noted that while Harris *et al.* [23] used *An. arabiensis* in their study, the present study used *An. gambiae* and the dosage of PPF was ten times higher than that of their study. Moreover the substrate of treatment in the previous study [23] was made of bottle while the present study, PPF was incorporated in bednet. Interestingly Brabant and Dobson [24] showed that methoprene could impair oviposition over two gonotrophic cycles in *Ae. aegypti* and larval breeding sites treatment with PPF could inhibit larval development up to five months [20].

A short contact time (three minutes) with PPF treated net and the long-residual effect of the chemical on mosquito reproductive output make it a very attractive control tool of malaria vectors. Most malaria vectors have developed resistance to pyrethroids and many other insecticides, threatening the future of vector control. Mixing PPF with pyrethroids on the nets can help control and limit the spread of resistance to pyrethroids in the distribution of malaria vectors. A couple of studies have already shown the potential of such mixtures raising the hope of a viable new vector control tool to help combat insecticide resistance [9,29]. Specifically previous studies with PPF hand-dipped nets demonstrated complete sterilization of laboratory reared pyrethroid susceptible *Anopheles gambiae* in bioassays. Further to this study, N’gufor *et al.* [9] have provided strong evidence that pyrethroid resistant *An. gambiae* were sterilized if they come to contact with bed nets that contain PPF. They concluded that a better reduction in vector abundance can be achieved with community wide use of Olyset Duo compared to LNs treated only with pyrethroids. In microcosms containing breeding sites to simulate the natural ecosystem, Ohba *et al.* [21] have shown that PPF had a strong sterilizing effect on *Aedes albopictus.*

According to current knowledge this is the first report of the effects of PPF-treated bed net on ovarian development in *An. gambiae*. In the present study, significantly higher retention rates of eggs and immature follicles were recorded in ovarioles when female mosquitoes were exposed to PPF compared to the controls. Combination of PPF and pyrethroids on bed nets may help to control pyrethroid-resistant mosquitoes because pyrethroid-resistant vectors that survive in contact with a net containing both insecticides could be sterilized by PPF. PPF presents a promising new opportunity for the integrated control of the main malaria vectors in sub-Saharan Africa.
